# Integrative mutation, haplotype and G × G interaction evidence connects ABGL4, LRP8 and PCSK9 genes to cardiometabolic risk

**DOI:** 10.1038/srep37375

**Published:** 2016-11-17

**Authors:** Tao Guo, Rui-Xing Yin, Li-Mei Yao, Feng Huang, Ling Pan, Wei-Xiong Lin, De-Zhai Yang, Shang-Ling Pan

**Affiliations:** 1 Department of Cardiology, Institute of Cardiovascular Diseases, the First Affiliated Hospital, Guangxi Medical University, Nanning 530021, Guangxi, People’s Republic of China; 2 Department of Molecular Genetics, Medical Scientific Research Center, Guangxi Medical University, Nanning 530021, Guangxi, People’s Republic of China; 3 Department of Pathophysiology, School of Premedical Sciences, Guangxi Medical University, Nanning 530021, Guangxi, People’s Republic of China

## Abstract

This study is expected to investigate the association of ATP/GTP binding protein-like 4 (AGBL4), LDL receptor related protein 8 (LRP8) and proprotein convertase subtilisin/kexin type 9 (PCSK9) gene single nucleotide variants (SNVs) with lipid metabolism in 2,552 individuals (Jing, 1,272 and Han, 1,280). We identified 12 mutations in this motif. The genotype and allele frequencies of these variants were different between the two populations. Multiple-locus linkage disequilibrium (LD) elucidated the detected sites are not statistically independent. Possible integrative haplotypes and gene-by-gene (G × G) interactions, comprising mutations of the *AGBL4*, *LRP8* and *PCSK9* associated with total cholesterol (TC, *AGBL4* G-G-A, *PCSK9* C-G-A-A and G-G-A-A-C-A-T-T-T-G-G-A), triglyceride (TG, *AGBL4* G-G-A, *LRP8* G-A-G-C-C, *PCSK9* C-A-A-G, A-A-G-G-A-G-C-C-C-A-A-G and A-A-G-G-A-G-C-C-C-G-A-A), HDL cholesterol (HDL-C, *AGBL4* A-A-G and A-A-G-A-A-G-T-C-C-A-A-G) and the apolipoprotein(Apo)A1/ApoB ratio (A1/B, *PCSK9* C-A-A-G) in Jing minority. However, in the Hans, with TG (*AGBL4* G-G-A, *LRP8* G-A-G-C-C, *PCSK9* C-A-A-G, A-A-G-G-A-G-C-C-C-A-A-G and A-A-G-G-A-G-C-C-C-G-A-A), HDL-C (*LRP8* A-A-G-T-C), LDL-C (*LRP8* A-A-G-T-C and A-A-G-A-A-G-T-C-C-A-A-G) and A1/B (*LRP8* A-C-A-T-T and *PCSK9* C-A-A-G). Association analysis based on haplotype clusters and G × G interactions probably increased power over single-locus tests especially for TG.

Cardiovascular disease (CVD) ranks as the leading cause of morbidity and mortality globally^1^, and increases extraordinarily in the developing country^2^. Cardiometabolic risk^3^,^4^ especially lipid metabolism dysfunction^5^ represents a key event in atherosclerosis, a pathogenesis of CVD. High total cholesterol (TC), triglyceride (TG), low-density lipoprotein cholesterol (LDL-C) and apolipoprotein (Apo) B concentrations, as well as low high-density lipoprotein cholesterol (HDL-C), ApoA1 levels and the ApoA1/ApoB ratio (A1/B) are considered as complex traits to which both genetic and environmental factors contribute^6^,^7^.

Despite hundreds of genome-wide hits from genome-wide association studies (GWAS), a large portion of variations in lipid metabolism attributable to heritability remains unexplained^8^. Because of stringent statistical cutoffs necessary in the GWAS methodology, it is argued many common variants with an appreciable effect on phenotypic variations are reported as false negatives and dismissed^9^. To correct away the hidden heritability, fine mapping follow from high-density replicated GWAS data need only use the tag single nucleotide variants (SNVs) and regions of linkage disequilibrium (LD) independent of annotation or relationship to nearby genes^10^.

Recently, the compelling genes for modifying lipid metabolism emerged from very large replicated GWAS: the ATP/GTP binding protein-like 4 gene (*AGBL4* [MIM 616476]), the LDL receptor related protein 8 gene (*LRP8* [MIM 602600]) and the proprotein convertase subtilisin/kexin type 9 gene (*PCSK9* [MIM 607786])^11^,^12^,^13^,^14^,^15^,^16^. The objective of this study was to perform association analysis to identify integrative mutations, haplotypes and gene-by-gene (G × G) interactions of the AGBL4 (rs320017 A > G, rs320018 A > G and rs320019 G > A), *LRP8* (rs6694764 G > A, rs1288519 A > C, rs872315 G > A, rs1288520 C > T and rs1288521 C > T) and *PCSK9* (rs533375 C > T, rs584626 A > G, rs585131 A > G and rs540796 G > A) associated with lipid phenotypic variations in the Jing and Han populations. Furthermore, we wanted to test if the association analysis of these loci based on haplotype clusters and G × G interactions increase power over single-locus tests.

## Results

### Study participants

Demographic, epidemiological and clinical characteristics of the 2, 552 analyzed study subjects are summarized in Table 1. The values of body mass index (BMI), waist circumference (WC) and the percentage of individuals whom consumed alcohol were higher, as well as the level of systolic blood pressure (SBP) was lower in Jing than Han (*P* < 0.05–0.001). For plasma lipid phenotypic variations, there were higher plasma TC and TG levels, as well as lower A1/B in Jing (*P* < 0.001, for each). However, no difference was noted in fasting plasma glucose, HDL-C and LDL-C levels between the two ethnic groups (*P* > 0.05 for all).

### Single-mutation association

**Figure 1:**
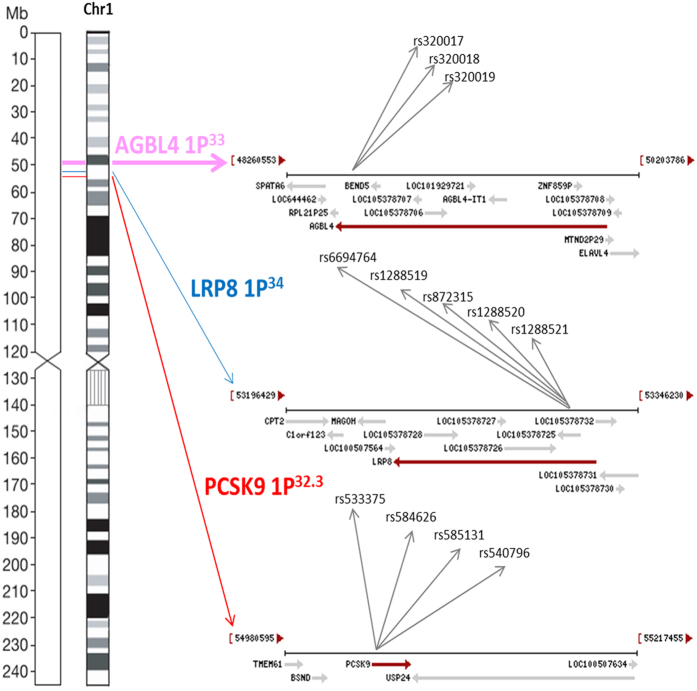
The positions of the *AGBL4*, *LRP8* and *PCSK9* mutations.

**Figure 2:**
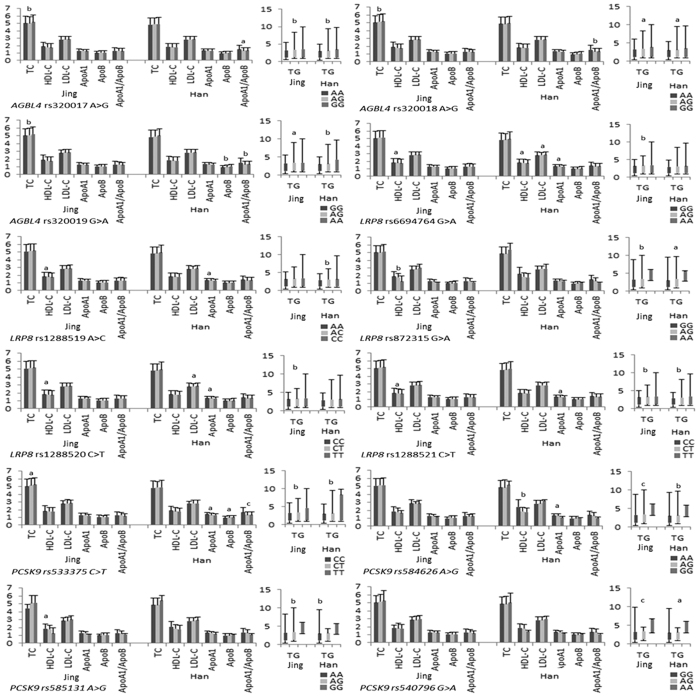
Single-mutation association with lipid phenotypic variations.

The detected 12 mutations in this motif are located in a closely genomic region of chromosome 1 (Fig. 1). As shown in Tables 2 and 3, the genotype and allele frequencies of these variants were different between the two populations (*P* < 0.05–0.001). All mutations exhibit the Hardy-Weinberg equilibrium (HWE, *P* > 0.05 for all). We tested each mutation individually for association with plasma lipid levels separately in each population. We discovered the association of the *AGBL4*, *LRP8* and *PCSK9* mutations with TC (rs320017, rs320018, rs320019 and rs533375), TG (rs320017, rs320018, rs320019, rs6694764, rs872315, rs1288520, rs1288521, rs533375, rs584626, rs585131 and rs540796) and HDL-C (rs6694764, rs1288519, rs872315, rs1288520, rs1288521 and rs585131) in Jing minority. However, in the Hans, with TG (rs320017, rs320018, rs320019, rs1288519, rs872315, rs1288521, rs533375, rs584626, rs585313 and rs540796), HDL-C (rs6694764 and rs584626), LDL-C (rs6694764 and rs1288520), ApoA1 (rs6694764, rs1288519, rs1288520, rs1288521, rs533375 and rs584626), ApoB (rs320019 and rs5333375) and A1/B (rs320017, rs320018, rs320019 and rs533375). (*P* < 0.05–0.001; Fig. 2).

### Haplotype-based association

**Figure 3:**
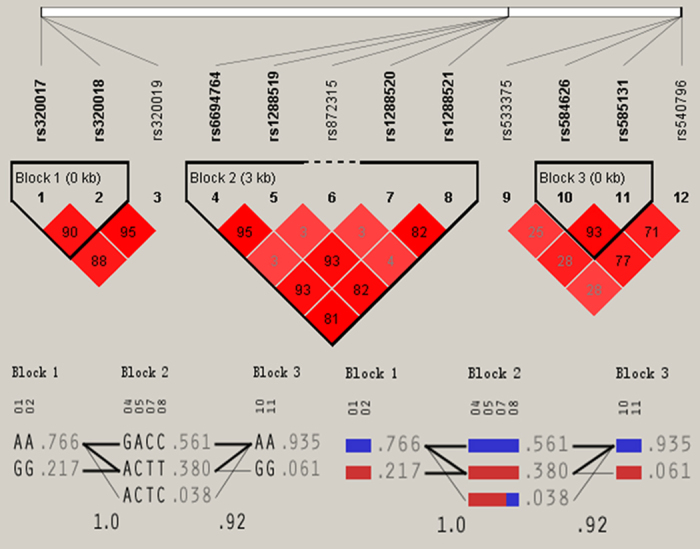
The LD plot represents pair-wise *r*^*2*^ and haplotypes frequency in the Jing population.

**Figure 4:**
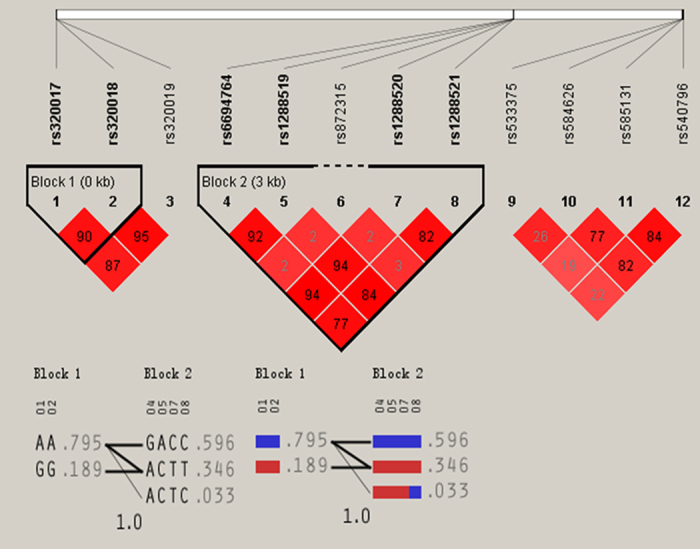
The LD plot represents pair-wise *r*^*2*^ and haplotypes frequency in the Han population.

**Figure 5:**
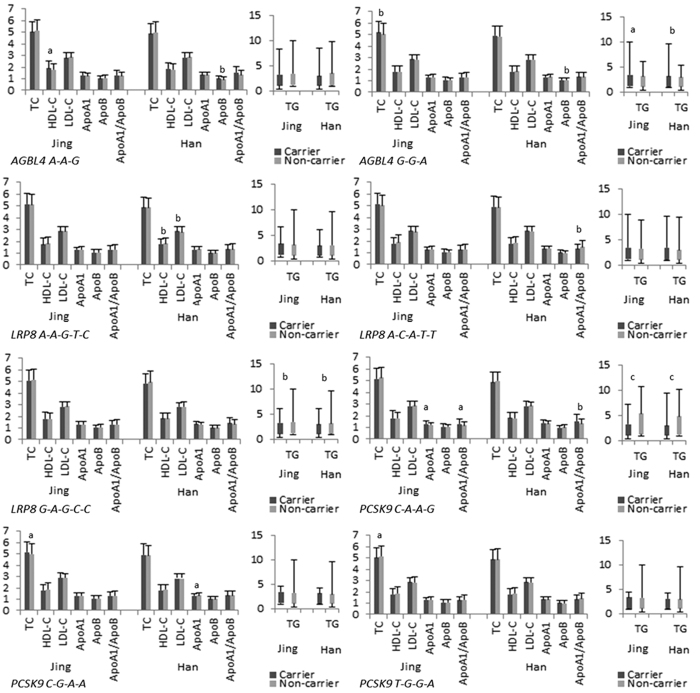
Haplotype-based association with lipid-related traits.

Multiple-locus linkage disequilibrium (LD) elucidated the detected sites were not statistically independent separately in each population. Figures 3 and 4 show the LD blocks and the haplotypes for blocks separately in the Jing and Han ethnic groups. As shown in Table 4, the commonest haplotypes were *AGBL4* A-A-G, *LRP8* G-A-G-C-C and *PCSK9* C-A-A-G (>50% of the samples). The frequencies of the *AGBL4* A-A-G, *AGBL4* G-G-A, *LRP8* A-A-G-T-C, *LRP8* A-C-A-T-T, *LRP8* G-A-G-C-C, *PCSK9* C-A-A-G, *PCSK9* C-G-A-A and *PCSK9* T-G-G-A haplotypes were quantitative significantly different between the Jing and Han populations (*P* < 0.05–0.001). We confirmed that the *AGBL4*, *LRP8* and *PCSK9* haplotypes were associated with TC (*AGBL4* G-G-A and *PCSK9* C-G-A-A), TG (*AGBL4* G-G-A, *LRP8* G-A-G-C-C and *PCSK9* C-A-A-G), HDL-C (*AGBL4* A-A-G), ApoA1 (*PCSK9* C-A-A-G), and A1/B (*PCSK9* C-A-A-G) in Jing minority. However, they were associated with TG (*AGBL4* G-G-A, *LRP8* G-A-G-C-C and *PCSK9* C-A-A-G), HDL-C (*LRP8* A-A-G-T-C), LDL-C (*LRP8* A-A-G-T-C), ApoA1 (*PCSK9* C-G-A-A), ApoB (*AGBL4* G-G-A) and A1/B (*LRP8* A-C-A-T-T and *PCSK9* C-A-A-G) in Han Chinese. (*P* < 0.05–0.001; Fig. 5).

### G × G interaction-based association

**Figure 6:**
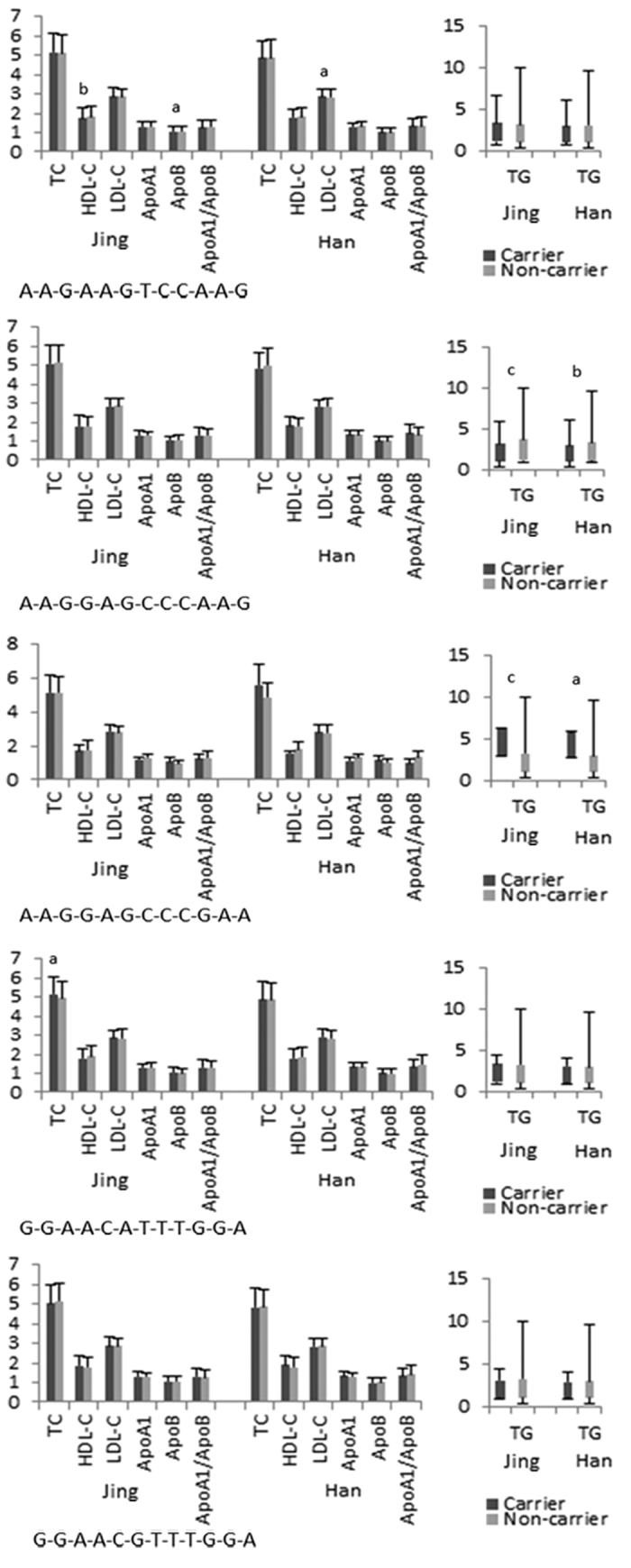
G × G interaction-based association with plasma lipid levels.

As shown in Table 5, the commonest G × G interaction was A-A-G-G-A-G-C-C-C-A-A-G (>50% of the samples). The frequencies of the A-A-G-A-A-G-T-C-C-A-A-G, A-A-G-G-A-G-C-C-C-A-A-G, A-A-G-G-A-G-C-C-C-G-A-A, G-G-A-A-C-A-T-T-T-G-G-A and G-G-A-A-C-G-T-T-T-G-G-A G × G interactions were significantly different between Jing and Han populations (*P* < 0.05–0.001). We identified that the G × G interactions among the detected mutations of *AGBL4*, *LRP8* and *PCSK9* were related with TC (G-G-A-A-C-A-T-T-T-G-G-A), TG (A-A-G-G-A-G-C-C-C-A-A-G and A-A-G-G-A-G-C-C-C-G-A-A), HDL-C (A-A-G-A-A-G-T-C-C-A-A-G) and ApoB (A-A-G-A-A-G-T-C-C-A-A-G) in Jing minority. However, in the Hans, with TG (A-A-G-G-A-G-C-C-C-A-A-G and A-A-G-G-A-G-C-C-C-G-A-A) and LDL-C (A-A-G-A-A-G-T-C-C-A-A-G). (*P* < 0.05–0.001; Fig. 6).

### Integrative association analysis of mutation, haplotype and G × G interaction

Table 6 depicts the integrative association analysis of mutation, haplotype and G × G interaction of *AGBL4*, *LRP8* and *PCSK9* with lipid phenotypic variations separately in the two ethnic groups. Generalized linear models adjusted for age, gender, BMI, WC, SBP, DBP, pulse pressure, cigarette smoking, alcohol consumption and fasting plasma glucose level demonstrated mutations, haplotypes and G × G interactions of *AGBL4*, *LRP8* and *PCSK9* quantitative significantly correlated with lipid-related traits. (*P* < 0.05–0.001). Furthermore, the association analysis based on haplotype clusters and G × G interactions probably increased power over single-locus tests especially for TG.

## Discussion

The main finding of the present study encompass (*i*) it elucidated the frequencies of mutation, haplotype and the G × G inter-locus interaction among AGBL4, LRP8 and PCSK9 genes in the Jing ethnic minority and Han population, which may be proposed as an potential supplement to the 1000 Genomes database (*ii*) it gave integrative mutation, haplotype and G × G interaction evidence to prove there are possible interaction between the AGBL4, LRP8 and PCSK9 genes and serum lipid concentrations; and (*iii*) it demonstrated association analysis based on haplotype clusters and G × G interactions probably increased power over single-locus tests especially for TG.

Aspects of primary prevention differ in some respects in ethnic minority groups when compared with general population^17^. Jing, as a group of migrants from Vietnam to south of China, maintains the higher cardiometabolic risk especially higher TC and TG, and lower A1/B ratio than local Han population living in the same natural and social environments. It is important to recognize that definitions of cardiometabolic risk especially dyslipidemia derived in local Han population perhaps inappropriate for ethnic minority groups. Resulting disease risks may remain difference in second and third generation migrants, even though blood pressure, fasting plasma glucose level and cigarette smoking lifestyle are converging towards those of the general Han population. Our present study pronounces differences in genetics values. The challenge now is to ensure that prevention and treatment services are ready to respond to these demographic and ethnic structure. Epidemiological survey has revealed that the Jing ethnic minority maintains genetic homogeneity. In the present study, all of the mutations satisfied with HWE separately in each population. It has been proved that the Jing and Han populations have different genetic ancestry from a statistical point of view. Our results showed that there was quantitative significantly different distributions of the detected 12 mutations of AGBL4, LRP8 and PCSK9 genes, their haplotypes and their G × G inter-locus interactions between the Jing and Han populations. These genetic heterogeneity may be correlated with the heterogeneousness of cardiometabolic risk especially dyslipidemia between the Jing and Han populations.

Environmental exposures cannot be ignored. We summarized the values of weight, BMI and WC were significantly different between the two populations. Maybe they are related with the custom of fish intake. Jing is an oceanic ethnic minority like Kinh populations in North Vietnam, survival relying on fishing^18^. Maybe there are differences in saturated fatty acid (SFA), polyunsaturated tatty acid (PUFA; n-3 PUFA and n-6 PUFA), and monounsaturated fatty acid (MUFA)^19^ intake to compare with the local Han population in their diet structure. Unfortunately it is only a hypothesis, because lack of dietary intake data. Consensus exists pertaining to the scientific evidence regarding effects of various those bad dietary fatty acids rich in fish on cardiometabolic risk including lipid phenotypic variations reported in a previous study^20^. What’s more, the cardiometabolic risk is known to be lower in light-to-moderate alcohol drinkers than in abstainers^21^. The effects of alcohol on lipid metabolism, especially the HDL cholesterol-elevating effects, are thought to greatly contribute to the cardio-protective action of alcohol^22^. On the other hand, excessive alcohol consumption has been shown to cause hypertriglyceridemia^23^,^24^, which is a prevalent risk factor for CVD. With regard to mechanisms underlying the effects of alcohol on lipid metabolism^25^,^26^,^27^, alcohol consumption has been shown to increase the activity of lipoprotein lipase and decrease the activity of cholesteryl ester transfer protein, resulting in elevation of HDL cholesterol^28^. Hypertriglyceridemia induced by excessive alcohol drinking may be mainly due to an increase in the synthesis of large very low-density lipoprotein (VLDL) particles in the liver. Consistently, the % of participants who consumed alcohol was different between the two groups. Wine culture plays a pivotal role in the history of China Han ethnic group. Many Han populations are good at alcohol consumption, especially in festivals.

Our data come from nuclear family and pedigree data, unfortunately, pedigree information were not documented. Heritability is a measure of familial resemblance^29^. Estimating the heritability of a trait represents one of the first steps in the gene mapping process. Or we can estimate heritability for quantitative traits from nuclear and pedigree data using the ASSOC program in the Statistical Analysis for Genetic Epidemiology (S.A.G.E.) software package. Estimating heritability rests on the assumption that the total phenotypic variance of a quantitative trait can be partitioned into independent genetic and environmental components^30^.

A number of clinical studies have demonstrated that inhibition of PCSK9 alone and in addition to statins potently reduces lipid phenotypic variation concentrations^31^,^32^. Plasma lipid phenotypic variation especially plasma TG level is heritable and modifiable^33^. Several groups have successfully to identify signals for TG and other lipid traits, including HDL-C, LDL-C, and TC^34^. However, the lead GWAS signals may not themselves be functional rather in LD with the actual underlying susceptibility mutations. The limitation in GWAS derives from the fact that the human genome is superficially screened using single independently tag SNVs. It is acknowledged that complex disease is not caused by or associated with one single variant. The functional mutation often acts through regional gene mutations, including haplotypes and G × G interactions. Therefore, GWAS, epigenome-wide association studies (EWAS) and transcriptome-wide association studies (TWAS) are only a starting and require subsequent fine mapping and functional validation to identify the actual susceptibility variants and gene interactions. AGBL4, LRP8 and PCSK9 genes are neighbors. Integrative mutations, haplotypes and G × G interactions evidence connects AGBL4, LRP8 and PCSK9 gene to lipid phenotypic variations perhaps can further elaborate the clinical application of PCSK9 inhibitors.

There are several limitations in our study. Firstly, the number of participants available for minor allele frequency (MAF) of some mutations was not high enough to calculate a strong power as compared with many previous GWAS and replication studies. Secondly, as an association analysis and observation study, inherent methodologic limitations that generate bias and confounding mean that causal inferences cannot reliably be drawn. Thirdly, take into consideration the randomized clinical trials (RCTs) provide the best opportunity to control for confounding and avoid certain biases. Consequently, well-designed, high-quality further therapeutic intervention study, including prophylactic agent, treatment, surgical approach, or diagnostic test is needed. Moreover, there are still many unmeasured environmental and genetic factors including TFA, SFA, PUFA (including n-3 PUFA and n-6 PUFA) and MUFA that needed to be considered. In addition, the relevance of this finding has to be defined in further high caliber of studies including incorporating the genetic information of AGBL4, LRP8 and PCSK9 gene mutations, haplotypes and G × G interactions *in vivo* and *vitro* functional studies to confirm the impact of a variant on a molecular level including transcription and expression. The last but not the least, discussion of race and ethnicity in medicine must rigorously avoid polarization and the further perpetuation of disparate health care.

In summary, there are potential interaction between the AGBL4, LRP8 and PCSK9 genes and serum lipid concentrations. And the association analysis based on haplotype clusters and G × G interactions probably increased power over single-locus tests especially for TG. These genetic heterogeneity may be correlated with the heterogeneousness of cardiometabolic risk between the Jing and Han populations. Differences in lipid phenotypic variations between the two populations might partially attribute to AGBL4, LRP8 and PCSK9 gene mutations, haplotypes and G × G interactions.

## Materials and Methods

### Ethical approval

The study were carried out following the rules of the Declaration of Helsinki of 1975 (http://www.wma.net/en/30publications/10policies/b3/), revised in 2008. All participants from contributing populations gave written informed consent to participate in epidemiologic investigation and genetic analysis. All study protocols in this motif have approval from the Ethics Committee of the First Affiliated Hospital, Guangxi Medical University (No: Lunshen-2011-KY-Guoji-001; Mar. 7, 2011).

### Subjects

Two groups of study population including 1272 unrelated participants of Jing (624 males, 49.06% and 648 females, 50.94%) and 1280 unrelated subjects of Han (636 males, 49.69% and 644 females, 50.31%) were randomly selected from our previous stratified randomized samples^35^. All participants were rural fishery (Jing) and/or agricultural (Han) workers from the three islands of Wanwei, Wutou and Shanxin in the county of Fangchenggang in the province of Guangxi, China, near the Sino-Vietnamese border. The participants’ age ranged from 18 to 80 years with a mean age of 57.27 ± 12.85 years in Jing and 56.85 ± 13.32 years in Han; respectively. The gender ratio and age distribution were matched between the two groups. All participants were essentially healthy with no history of coronary artery disease, stroke, diabetes, hyper- or hypo-thyroids, and chronic renal disease. They were free from medications known to affect lipid profiles.

### Epidemiological survey

The epidemiological survey was carried out using internationally standardized method, following a common protocol^36^. Information on demographics, socioeconomic status, and lifestyle factors were collected with standardized questionnaires. Cigarette smoking status was categorized into groups of cigarettes per day: ≤20 and >20^37^. Alcohol consumption was categorized into groups of grams of alcohol per day: ≤25 and >25^38^. Several parameters such as blood pressure, height, weight and WC were measured, while BMI (kg/m^2^) was calculated. BMI was categorized into four groups: underweight (BMI < 18.5), normal weight (18.5 ≤ BMI < 24), overweight (24 ≤ BMI < 28) and Obesity (28 ≤ BMI)^39^. Likewise, WC was categorized into groups including normal group (WC ≤ 85 for male and WC ≤ 80 for female) and abdominal obesity (WC > 85 for male and WC > 80 for female)^40^.

### Biochemical measurements

A fasting venous blood sample of 5 ml was drawn from the participants. The levels of fasting plasma TC, TG, HDL-C and LDL-C in the samples were determined by enzymatic methods with commercially available kits. Fasting plasma ApoA1 and ApoB levels were assessed by the immuneturbidimetric immunoassay.

### Diagnostic criteria

The normal values of fasting plasma TC, TG, HDL-C, LDL-C, ApoA1 and ApoB levels, as well as the A1/B ratio in our Clinical Science Experiment Center were 3.10–5.17, 0.56–1.70, 1.16–1.42, 2.70–3.10 mmol/L, 1.20–1.60, 0.80–1.05 g/L, and 1.00–2.50; respectively^41^.

### Mutation selection

We selected 12 mutations in the *AGBL4*, *LRP8* and *PCSK9* with the following assumption: (i) tag SNVs, which were established by Haploview (Broad Instituteof MIT and Harvard, Cambridge, MA, USA, version 4.2); (ii) functional mutations (http://snpinfo.niehs.nih.gov/snpinfo/snpfunc.htm) in functional areas of the gene fragment from NCBI dbSNP Build 132 (http://www-ncbi-nlm-nih-gov.ezp-prod1.hul.harvard.edu/SNP/); (iii) a known minor allele frequency (MAF) higher than 1% in European ancestry from the Human Genome Project Database; and (iiii) mutations might be associated with the lipid-related traits or cardiometabolic risk in the latest studies.

### Genotyping

Genomic DNA was extracted from leucocytes of venous blood using the phenol-chloroform method. Genotyping of 12 mutations was performed by PCR and Sanger sequencing. The characteristics of each mutation and the details of each primer pair, annealing temperature, length of the PCR products are summarized in Supplemental Tables 1 and 2. The PCR products of the samples were sequenced with a sequencer ABI Prism 3100 Genetic Analyzer (Applied Biosystems, International Equipment Trading Ltd., Vernon Hills, IL, USA) in Shanghai Sangon Biological Engineering Technology & Services Co. Ltd., Shanghai China.

### Statistical Analyses

The statistical analysis were performed with the statistical software SPSS 21.0 (SPSS Inc., Chicago, IL, USA). Quantitative variables were presented as the mean ±SD for those, that are normally distributed, whereas the medians and interquartile ranges for TG, which is not normally distributed. General characteristics between the two groups were compared by the *ANCOVA*. The distributions of the genotype, allele, haplotype and G × G interaction between the two groups were analyzed by the chi-squared test; The HWE, Pair-wise LD, frequencies of haplotype and G × G interaction comprising the mutations were calculated using Haploview (version 4.2; Broad Institute of MIT and Harvard). The association of the genotypes, haplotypes and G × G interactions with lipid phenotypic variations was tested by the *Univariant*. Any variants associated with the lipid phenotypic variations at a value of *P* < 0.05 were considered statistically significant. Generalized linear models were used to assess the association of the genotypes (common homozygote genotype = 1, heterozygote genotype = 2, rare homozygote genotype = 3), alleles (the minor allele non-carrier = 1, the minor allele carrier = 2), haplotypes (the haplotype non-carrier = 1, the haplotype carrier = 2) and G × G interactions (the G × G interaction non-carrier = 1, the G × G interaction carrier = 2) with lipid phenotypic variations. The model of age, gender, BMI, WC, SBP, DBP, pulse pressure, cigarette smoking, alcohol consumption and fasting plasma glucose level were adjusted for the statistical analysis. The pattern of pair-wise LD between the selected mutations was measured by *D*′ and *r*^*2*^ using the Haploview software.

## Additional Information

**How to cite this article**: Guo, T. *et al.* Integrative mutation, haplotype and G × G interaction evidence connects ABGL4, LRP8 and PCSK9 genes to cardiometabolic risk. *Sci. Rep.* **6**, 37375; doi: 10.1038/srep37375 (2016).

**Publisher’s note:** Springer Nature remains neutral with regard to jurisdictional claims in published maps and institutional affiliations.

## Supplementary Material

Supplementary Information

## Figures and Tables

**Table 1:** Demographic, epidemiological and clinical characteristics.

Characteristics	Jing	Han	*test-statistic*	*P-value*
Number (n)	1272	1280		
Gender (Male/Female)	624/648	636/644	0.102	0.750
Age (years)^1^	57.27 ± 12.85	56.85 ± 13.32	0.818	0.414
Height (cm)	158.51 ± 7.93	157.63 ± 8.02	2.796	0.005
Weight (kg)	58.88 ± 10.03	56.78 ± 9.36	5.489	4.453E-08
Body mass index (kg/m^2^)	23.37 ± 3.17	22.82 ± 3.16	4.405	1.101E-05
Underweight(BMI < 18.5)	52(4.1)	90(7.0)		
Normal weight(18.5 ≤ BMI < 24)	729(57.3)	761(59.5)		
Overweight(24 ≤ BMI < 28)	387(30.4)	356(27.8)		
Obesity(28 ≤ BMI)	104(8.2)	73(5.7)	17.554	0.001
Waist circumference (cm)	80.24 ± 9.26	77.93 ± 8.72	6.484	1.073E-10
Male(Waist circumference ≤85)	398(63.8)	520(81.8)		
Male(Waist circumference >85)	226(36.2)	116(18.2)	51.484	7.218E-13
Female(Waist circumference ≤80)	376(58.0)	412(64.3)		
Female(Waist circumference >80)	272(42.0)	229(35.7)	5.297	0.021
Systolic blood pressure (mmHg)	131.37 ± 20.92	134.84 ± 29.18	−1.968	0.049
Diastolic blood pressure (mmHg)	80.81 ± 10.55	81.05 ± 10.29	−0.561	0.575
Pulse pressure (mmHg)	50.56 ± 16.84	53.79 ± 27.63	−1.920	0.055
Cigarette smoking [n (%)]				
Nonsmoker	1008(79.25)	989(77.26)		
≤20 Cigarette smoking/day	63(4.95)	59(4.61)		
>20 Cigarette smoking/day	201(15.80)	232(18.13)	2.506	0.286
Alcohol consumption [n (%)]				
Nondrinker	971(76.34)	870(67.97)		
≤25 g/day	157(12.34)	90(7.03)		
>25 g/day	144(11.32)	320(25.00)	90.450	2.286E-20
Blood glucose level (mmol/L)	6.70 ± 1.71	6.63 ± 1.11	1.353	0.176
Total cholesterol (mmol/L)	5.15 ± 0.91	4.88 ± 0.85	7.877	4.935E-15
Triglyceride (mmol/L)^2^	1.43(1.12)	1.32(1.07)	−4.439	9.018E-06
High-density lipoprotein cholesterol (mmol/L)	1.78 ± 0.53	1.81 ± 0.46	−1.544	0.123
Low-density lipoprotein cholesterol (mmol/L)	2.86 ± 0.43	2.83 ± 0.43	1.477	0.140
Apolipoprotein (Apo) A1 (g/L)	1.31 ± 0.24	1.33 ± 0.20	−2.305	0.021
ApoB (g/L)	1.06 ± 0.25	1.04 ± 0.24	2.693	0.007
ApoA1/ApoB	1.30 ± 0.38	1.35 ± 0.38	−3.528	4.256E-04

^1^Mean ± SD determined by *t*-test.

^2^Median (interquartile range) tested by the *Wilcoxon-Mann-Whitney* test.

**Table 2:** Prevalence of genotype frequencies in the Jing and Han populations [n (%)].

Mutation	Genotype	Jing (n = 1272)	Han (n = 1280)	X^2^	*P*-value
*AGBL4* rs320017 A > G	AA	768(60.38)	836(65.31)	6.704	0.035
	AG	438(34.43)	388(30.31)		
	GG	66(5.19)	56(4.38)		
	*P*_HWE_	0.730	0.202		
*AGBL4* rs320018 A > G	AA	762(59.90)	827(64.61)	6.137	0.046
	AG	443(34.83)	397(31.01)		
	GG	67(5.27)	56(4.82)		
	*P*_HWE_	0.802	0.344		
*AGBL4* rs320019 G > A	GG	769(60.46)	834(65.16)	6.054	0.048
	AG	438(34.43)	387(30.23)		
	AA	65(5.11)	59(4.61)		
	*P*_HWE_	0.797	0.105		
*LRP8* rs6694764 G > A	GG	405(31.84)	465(36.33)	6.657	0.036
	AG	632(49.69)	611(47.73)		
	AA	235(18.47)	204(15.94)		
	*P*_HWE_	0.674	0.889		
*LRP8* rs1288519 A > C	AA	425(33.41)	484(37.81)	6.270	0.043
	AC	618(48.59)	597(46.64)		
	CC	229(18.00)	199(15.55)		
	*P*_HWE_	0.868	0.507		
*LRP8* rs872315 G > A	GG	1180(92.77)	1224(95.63)	9.588	0.008
	AG	88(6.92)	54(4.21)		
	AA	4(0.31)	2(0.16)		
	*P*_HWE_	0.091	0.090		
*LRP8* rs1288520 C > T	CC	431(33.88)	502(39.22)	10.453	0.005
	CT	590(46.38)	574(44.84)		
	TT	251(19.74)	204(15.94)		
	*P*_HWE_	0.057	0.064		
*LRP8* rs1288521 C > T	CC	490(38.52)	554(43.28)	6.169	0.046
	CT	587(46.15)	552(43.13)		
	TT	195(15.33)	174(13.59)		
	*P*_HWE_	0.356	0.053		
*PCSK9* rs533375 C > T	CC	886(69.65)	963(75.23)	10.534	0.005
	CT	340(26.73)	285(22.27)		
	TT	46(3.62)	32(2.50)		
	*P*_HWE_	0.064	0.051		
*PCSK9* rs584626 A > G	AA	1116(87.74)	1162(90.78)	6.649	0.036
	AG	148(11.63)	114(8.91)		
	GG	8(0.63)	4(0.31)		
	*P*_HWE_	0.207	0.501		
*PCSK9* rs585131 A > G	AA	1118(87.89)	1172(91.56)	9.332	0.009
	AG	150(11.79)	105(8.20)		
	GG	4(0.32)	3(0.23)		
	*P*_HWE_	0.663	0.689		
*PCSK9* rs540796 G > A	GG	1092(85.85)	1159(90.55)	15.222	4.949E-04
	AG	172(13.52)	119(9.30)		
	AA	8(0.63)	2(0.15)		
	*P*_HWE_	0.666	0.560		

*AGBL4*, the ATP/GTP binding protein-like 4 gene; *LRP8*, the LDL receptor related protein 8 gene; *PCSK9*, the Proprotein convertase subtilisin/kexin type 9 gene; HWE, Hardy-Weinberg equilibrium.

**Table 3:** Prevalence of allele frequencies in the Jing and Han populations [n(%)].

Mutation	Allele	Jing (n = 1272)	Han (n = 1280)	X^2^	P-value
*AGBL4* rs320017	A/G	1974(77.59)/570(22.41)	2060(80.47)/500(19.53)	6.363	0.012
*AGBL4* rs320018	A/G	1967(77.32)/577(22.68)	2051(80.12)/509(19.88)	5.964	0.015
*AGBL4* rs320019	G/A	1976(77.67)/568(22.33)	2055(80.27)/505(19.73)	5.197	0.023
*LRP8* rs6694764	G/A	1442(56.68)/1102(43.32)	1541(60.20)/1019(39.80)	6.484	0.011
*LRP8* rs1288519	A/C	1468(57.70)/1076(42.30)	1565(61.13)/995(38.87)	6.220	0.013
*LRP8* rs872315	G/A	2448(96.23)/96(3.77)	2502(97.73)/58(2.27)	9.480	0.002
*LRP8* rs1288520	C/T	1452(57.08)/1092(42.92)	1578(61.64)/982(38.36)	11.024	0.001
*LRP8* rs1288521	C/T	1567(61.60)/977(38.40)	1660(64.84)/900(35.16)	5.789	0.016
*PCSK9* rs533375	C/T	2112(83.02)/432(16.98)	2211(86.37)/349(13.63)	11.038	0.001
*PCSK9* rs584626	A/G	2380(93.55)/164(6.45)	2438(95.23)/122(4.77)	6.816	0.009
*PCSK9* rs585131	A/G	2386(93.79)/158(6.21)	2449(95.66)/111(4.34)	8.983	0.003
*PCSK9* rs540796	G/A	2356(92.61)/188(7.39)	2437(95.20)/123(4.80)	14.904	1.131E-04

*AGBL4*, the ATP/GTP binding protein-like 4 gene; *LRP8*, the LDL receptor related protein 8 gene; *PCSK9*, the Proprotein convertase subtilisin/kexin type 9 gene.

**Table 4:** Prevalence of haplotype frequencies in the Jing and Han populations [n (frequency)].

Haplotype	Jing	Han	X^2^	*P*-value	Odds Ratio [95%CI]
*AGBL4* A-A-A	0.00(0.000)	4.95(0.002)	4.927	0.026456	—
*AGBL4* A-A-G	1948.87(0.766)	2030.84(0.793)	5.509	0.018941	0.853 [0.747~0.974]
*AGBL4* A-G-A	24.62(0.010)	24.21(0.009)	0.007	0.935631	1.023 [0.582~1.798]
*AGBL4* G-A-A	5.12(0.002)	3.15(0.001)	0.481	0.487924	1.636 [0.402~6.663]
*AGBL4* G-A-G	13.01(0.005)	12.06(0.005)	0.043	0.836051	1.086 [0.495~2.383]
*AGBL4* G-G-A	538.26(0.212)	472.69(0.185)	5.830	0.015777	1.185 [1.032~1.360]
*AGBL4* G-G-G	13.60(0.005)	12.10(0.005)	0.098	0.754777	1.132 [0.521~2.460]
*AGBL4* A-G-G	0.51(0.000)	0.00(0.000)	0.517	0.472063	—
*LRP8* A-A-G-C-C	13.00(0.005)	24.19(0.009)	3.322	0.068348	0.538 [0.274~1.059]
*LRP8* A-A-G-T-C	3.03(0.001)	12.06(0.005)	5.361	0.020611	0.252 [0.071~0.889]
*LRP8* A-C-A-T-T	88.00(0.035)	54.00(0.021)	8.594	0.003384	1.663 [1.180~2.344]
*LRP8* A-C-G-C-C	12.08(0.005)	12.01(0.005)	0.001	0.975872	1.012 [0.455~2.254]
*LRP8* A-C-G-C-T	0.00(0.000)	0.80(0.000)	0.791	0.373701	—
*LRP8* A-C-G-T-C	96.89(0.038)	84.86(0.033)	0.906	0.341247	1.155 [0.858~1.554]
*LRP8* A-C-G-T-T	879.03(0.346)	831.08(0.325)	2.499	0.113912	1.098 [0.978~1.234]
*LRP8* G-A-A-C-C	8.00(0.003)	4.00(0.002)	1.361	0.243302	2.015 [0.606~6.700]
*LRP8* G-A-G-C-C	1417.91(0.557)	1522.66(0.595)	7.322	0.006827	0.858 [0.768~0.959]
*LRP8* G-A-G-C-T	0.00(0.000)	2.09(0.001)	2.074	0.149794	—
*LRP8* G-C-G-C-C	0.00(0.000)	0.22(0.000)	0.219	0.639983	—
*LRP8* G-C-G-C-T	0.00(0.000)	12.03(0.005)	11.985	0.000540	—
*LRP8* A-A-G-C-T	1.01(0.000)	0.00(0.000)	1.014	0.313942	—
*LRP8* A-A-G-T-T	8.96(0.004)	0.00(0.000)	9.032	0.002663	—
*LRP8* G-A-G-T-C	16.09(0.006)	0.00(0.000)	16.243	5.63e-005	—
*PCSK9* C-A-A-A	9.15(0.004)	3.01(0.001)	3.150	0.075953	3.069 [0.833~11.303]
*PCSK9* C-A-A-G	2086.39(0.820)	2191.61(0.856)	12.173	0.000488	0.766 [0.660~0.890]
*PCSK9* C-A-G-A	0.00(0.000)	8.02(0.003)	7.978	0.004749	—
*PCSK9* C-G-A-A	8.01(0.003)	0.51(0.000)	5.953	0.014713	15.703 [2.003~123.131]
*PCSK9* C-G-A-G	0.00(0.000)	0.54(0.000)	0.539	0.462699	—
*PCSK9* C-G-G-A	4.42(0.002)	3.86(0.002)	0.042	0.836807	1.154 [0.294~4.528]
*PCSK9* C-G-G-G	4.03(0.002)	3.46(0.001)	0.048	0.827146	1.173 [0.279~4.941]
*PCSK9* T-A-A-G	259.47(0.102)	235.37(0.092)	1.472	0.224998	1.122 [0.932~1.351]
*PCSK9* T-G-A-A	0.00(0.000)	11.93(0.005)	11.888	0.000569	—
*PCSK9* T-G-A-G	0.00(0.000)	6.02(0.002)	5.993	0.014383	—
*PCSK9* T-G-G-A	143.43(0.056)	95.67(0.037)	10.327	0.001317	1.539 [1.181~2.006]
*PCSK9* T-A-A-A	22.99(0.009)	0.00(0.000)	23.237	1.46e-006	—
*PCSK9* T-A-G-G	2.00(0.001)	0.00(0.000)	2.018	0.155421	—
*PCSK9* T-G-G-G	4.10(0.002)	0.00(0.000)	4.133	0.042083	—

*AGBL4*, the ATP/GTP binding protein-like 4 gene; *LRP8*, the LDL receptor related protein 8 gene; *PCSK9*, the Proprotein convertase subtilisin/kexin type 9 gene.

**Table 5:** Prevalence of G × G interaction frequencies in the Jing and Han populations [n (frequency)].

G × G interactions												Jing	Han	X^2^	*P*-value	Odds Ratio[95%CI]
A	B	C	D	E	F	G	H	I	J	K	L					
A	A	G	A	A	G	C	C	C	A	A	G	13.00(0.005)	24.20(0.009)	3.325	0.068240	1.858 [0.945~3.653]
A	A	G	A	A	G	C	T	C	A	A	G	1.01(0.000)	0.00(0.000)	1.014	0.313942	—
A	A	G	A	A	G	T	C	C	A	A	G	4.00(0.002)	12.07(0.005)	4.015	0.045102	3.009 [0.970~9.340]
A	A	G	A	A	G	T	T	C	A	A	G	7.99(0.003)	0.00(0.000)	8.054	0.004555	—
A	A	G	A	G	G	C	C	C	A	A	G	12.09(0.005)	12.00(0.005)	0.001	0.973787	0.987 [0.443~2.197]
A	A	G	A	C	G	T	C	C	A	A	G	95.90(0.038)	84.09(0.033)	0.882	0.347570	0.867 [0.643~1.168]
A	A	G	A	C	G	T	T	C	A	A	G	369.92(0.145)	356.86(0.139)	0.377	0.539053	0.952 [0.814~1.114]
A	A	G	A	C	G	T	T	T	A	A	G	3.08(0.001)	0.00(0.000)	3.097	0.078447	—
A	A	G	G	A	A	C	C	C	G	G	A	4.00(0.002)	2.00(0.001)	0.680	0.409551	0.496 [0.091~2.713]
A	A	G	G	A	A	C	C	C	G	G	G	4.00(0.002)	2.00(0.001)	0.680	0.409551	0.496 [0.091~2.713]
A	A	G	G	A	G	C	C	C	A	A	A	8.00(0.003)	3.00(0.001)	2.309	0.128579	0.372 [0.099~1.403]
A	A	G	G	A	G	C	C	C	A	A	G	1401.90(0.551)	1507.63(0.589)	7.460	0.006325	1.167 [1.045~1.304]
A	A	G	G	A	G	C	C	C	G	A	A	8.00(0.003)	0.50(0.000)	5.982	0.014474	0.062 [0.008~0.492]
A	A	G	G	A	G	T	C	C	A	A	G	16.10(0.006)	0.00(0.000)	16.248	5.61e-005	—
A	G	A	A	C	A	T	T	T	G	G	A	12.01(0.005)	12.00(0.005)	0.000	0.985825	0.993 [0.445~2.214]
A	G	A	A	C	G	T	T	T	A	A	G	12.00(0.005)	12.10(0.005)	0.000	0.996678	1.002 [0.450~2.230]
A	G	G	A	C	G	T	T	T	A	A	G	1.00(0.000)	0.00(0.000)	1.006	0.315782	—
G	A	A	A	C	G	T	T	C	A	A	G	5.00(0.002)	3.10(0.001)	0.459	0.498128	0.616 [0.149~2.541]
G	A	G	A	C	A	T	T	T	G	G	A	11.29(0.004)	0.00(0.000)	11.389	0.000743	—
G	A	G	A	C	G	T	T	C	A	A	G	1.72(0.001)	0.00(0.000)	1.734	0.187919	—
G	G	A	A	C	A	T	T	T	G	G	A	60.53(0.024)	36.00(0.014)	6.513	0.010727	0.585 [0.386~0.887]
G	G	A	A	C	A	T	T	T	G	G	G	4.16(0.002)	0.00(0.000)	4.194	0.040582	—
G	G	A	A	C	G	T	T	C	A	A	G	147.37(0.058)	160.01(0.063)	0.472	0.492153	1.084 [0.861~1.366]
G	G	A	A	C	G	T	T	T	A	A	A	24.16(0.009)	0.00(0.000)	24.431	7.84e-007	—
G	G	A	A	C	G	T	T	T	A	A	G	241.74(0.095)	222.87(0.087)	0.979	0.322457	0.908 [0.750~1.099]
G	G	A	A	C	G	T	T	T	A	G	G	1.00(0.000)	0.00(0.000)	1.006	0.315782	—
G	G	A	A	C	G	T	T	T	G	G	A	60.00(0.024)	36.00(0.014)	6.270	0.012298	0.590 [0.389~0.896]
G	G	G	A	C	G	T	T	C	A	A	G	12.00(0.005)	12.00(0.005)	0.000	0.987907	0.994 [0.446~2.216]
G	G	G	A	C	G	T	T	T	A	G	G	1.00(0.000)	0.00(0.000)	1.006	0.315782	—
A	A	A	A	C	G	T	T	C	A	A	G	0.00(0.000)	4.90(0.002)	4.874	0.027288	—
A	A	G	A	C	G	C	T	C	A	A	G	0.00(0.000)	0.80(0.000)	0.799	0.371406	—
A	A	G	G	A	G	C	C	C	A	G	A	0.00(0.000)	8.00(0.003)	7.962	0.004789	—
A	A	G	G	A	G	C	C	C	G	A	G	0.00(0.000)	0.50(0.000)	0.496	0.481221	—
A	A	G	G	A	G	C	C	C	G	G	A	0.00(0.000)	1.50(0.001)	1.491	0.222007	—
A	A	G	G	A	G	C	C	C	G	G	G	0.00(0.000)	1.50(0.001)	1.491	0.222007	—
A	A	G	G	A	G	C	T	C	A	A	G	0.00(0.000)	2.10(0.001)	2.089	0.148303	—
A	A	G	G	C	G	C	C	C	A	A	G	0.00(0.000)	1.01(0.000)	1.008	0.315460	—
A	A	G	G	C	G	C	T	C	A	A	G	0.00(0.000)	11.23(0.004)	11.180	0.000831	—
G	A	G	A	C	G	T	T	T	G	G	A	0.00(0.000)	12.00(0.005)	11.952-	0.000549	—
G	G	A	A	C	A	T	T	T	G	A	G	0.00(0.000)	6.00(0.002)	5.970	0.014571	—
G	G	A	A	C	G	T	T	T	G	A	A	0.00(0.000)	12.00(0.005)	11.952	0.000549	—
G	G	A	G	C	G	C	T	T	A	A	G	0.00(0.000)	0.03(0.000)	0.028	0.867129	—

A, *AGBL4* rs320017 A > G; B, *AGBL4* rs320018 A > G; C, *AGBL4* rs320019 G > A; D, *LRP8* rs6694764 G > A; E, *LRP8* rs1288519 A > C; F, *LRP8* rs872315 G > A; G, *LRP8* rs1288520 C > T; H, *LRP8* rs1288521 C > T; I, *PCSK9* rs533375 C > T; J, *PCSK9* rs584626 A > G;K, *PCSK9* rs585131 A > G;L, *PCSK9* rs540796 G > A; *AGBL4*, the ATP/GTP binding protein-like 4 gene; *LRP8*, the LDL receptor related protein 8 gene; *PCSK9*, the Proprotein convertase subtilisin/kexin type 9 gene.

**Table 6:** Association of integrative *AGBL4*, *LRP8* and *PCSK9* mutations, haplotypes and G × G interactions with lipid-related traits in the Jing and Han populations.

Lipid	Mutation/Hapolype/G × G interaction	Affected phenotype/Other phenotype	Unstandardized Coefficients		Standardized Coefficients	*t*	*P*-value
			B	Std.error	Beta		
Jing							
TC	*PCSK9* rs533375	CC/CT/TT	0.194	0.090	0.117	2.160	0.031
	*PCSK9* rs585131	A/G	0.823	0.363	0.296	2.270	0.023
	*AGBL4* G-G-A	Carriers/Non-carriers	0.167	0.050	0.089	3.368	0.001
	*PCSK9* C-G-A-A	Carriers/Non-carriers	0.178	0.085	0.056	2.100	0.036
	*PCSK9* T-G-G-A	Carriers/Non-carriers	0.151	0.076	0.053	1.992	0.047
	G-G-A-A-C-A-T-T-T-G-G-A	Carriers/Non-carriers	0.234	0.104	0.059	2.236	0.026
TG	*AGBL4* rs320017	AA/AG/GG	0.272	0.099	0.174	2.752	0.006
	*AGBL4* rs320017	A/G	0.894	0.205	0.473	4.369	1.352E-05
	*AGBL4* rs320018	A/G	−1.287	0.244	−0.683	−5.274	1.573E-07
	*AGBL4* rs320019	G/A	0.533	0.208	0.282	2.556	0.011
	*LRP8* rs6694764	GG/AG/AA	0.980	0.165	0.739	5.933	3.845E-09
	*LRP8* rs6694764	G/A	0.623	0.190	0.314	3.283	0.001
	*LRP8* rs1288519	AA/AC/CC	−1.588	0.171	−1.203	−9.278	7.367E-20
	*LRP8* rs1288519	A/C	−1.048	0.224	−0.535	−4.675	3.265E-06
	*LRP8* rs1288520	CC/CT/TT	0.537	0.141	0.417	3.819	1.405E-04
	*LRP8* rs1288520	C/T	0.437	0.219	0.224	1.991	0.047
	*LRP8* rs1288521	CC/CT/TT	0.226	0.088	0.171	2.576	0.010
	*PCSK9* rs533375	CC/CT/TT	0.348	0.081	0.205	4.301	1.831E-05
	*PCSK9* rs584626	AA/AG/GG	0.724	0.182	0.277	3.986	7.113E-05
	*PCSK9* rs540796	GG/AG/AA	−0.621	0.118	−0.250	−5.276	1.557E-07
	*PCSK9* rs540796	G/A	−0.754	0.146	−0.284	−5.163	2.829E-07
	*AGBL4* A-A-G	Carriers/Non-carriers	0.357	0.096	0.093	3.723	2.052E-04
	*AGBL4* G-G-A	Carriers/Non-carriers	−0.234	0.047	−0.123	−4.979	0.000
	*LRP8* G-A-G-C-C	Carriers/Non-carriers	0.349	0.057	0.151	6.110	1.329E-09
	*PCSK9* C-A-A-G	Carriers/Non-carriers	1.110	0.105	0.255	10.577	4.166E-25
	A-A-G-G-A-G-C-C-C-A-A-G	Carriers/Non-carriers	0.400	0.056	0.175	7.149	1.482E-12
	A-A-G-G-A-G-C-C-C-G-A-A	Carriers/Non-carriers	−1.302	0.335	−0.097	−3.888	1.065E-04
HDL-C	*LRP8* rs6694764	GG/AG/AA	0.221	0.112	0.291	1.977	0.048
	*LRP8* rs1288519	AA/AC/CC	−0.236	0.116	−0.313	−2.040	0.042
	*AGBL4* A-A-G	Carriers/Non-carriers	0.123	0.061	0.056	2.016	0.044
	*LRP8* A-A-G-T-C	Carriers/Non-carriers	0.071	0.029	0.067	2.414	0.016
	A-A-G-A-A-G-T-C-C-A-A-G	Carriers/Non-carriers	0.082	0.029	0.078	2.792	0.005
LDL-C	*LRP8* rs1288521	CC/CT/TT	0.108	0.049	0.177	2.226	0.026
ApoA1	*PCSK9* C-A-A-G	Carriers/Non-carriers	−0.075	0.031	−0.066	−2.417	0.016
ApoB	*LRP8* rs1288520	CC/CT/TT	0.091	0.044	0.262	2.060	0.040
	*LRP8* rs1288521	CC/CT/TT	0.061	0.028	0.171	2.220	0.027
	*LRP8* rs1288521	C/T	0.090	0.034	0.176	2.618	0.009
	A-A-G-A-A-G-T-C-C-A-A-G	Carriers/Non-carriers	−0.028	0.014	−0.056	−2.050	0.041
ApoA1/ApoB	*LRP8* rs1288521	C/T	−0.121	0.052	−0.154	−2.311	0.021
	*PCSK9* C-A-A-G	Carriers/Non-carriers	−0.098	0.049	−0.054	−2.004	0.045
Han							
TC	*AGBL4* rs320018	AA/AG/GG	−0.551	0.209	−0.370	−2.632	0.009
	*AGBL4* rs320019	GG/AG/AA	0.505	0.187	0.341	2.698	0.007
TG	*AGBL4* rs320017	AA/AG/GG	0.543	0.117	0.311	4.635	3.942E-06
	*AGBL4* rs320017	A/G	1.059	0.253	0.507	4.178	3.147E-05
	*AGBL4* rs320018	AA/AG/GG	−1.793	0.213	−1.030	−8.416	1.049E-16
	*AGBL4* rs320018	A/G	−1.447	0.300	-.695	−4.826	1.562E-06
	*AGBL4* rs320019	GG/AG/AA	1.262	0.190	0.730	6.628	5.035E-11
	*LRP8* rs6694764	GG/AG/AA	−0.446	0.153	−0.311	−2.906	0.004
	*LRP8* rs1288519	AA/AC/CC	1.173	0.160	0.820	7.334	4.005E-13
	*LRP8* rs1288519	A/C	0.540	0.249	0.263	2.173	0.030
	*LRP8* rs872315	GG/AG/AA	0.489	0.176	0.106	2.779	0.006
	*LRP8* rs872315	G/A	0.616	0.181	0.127	3.412	0.001
	*LRP8* rs1288520	CC/CT/TT	−1.049	0.176	−0.743	−5.974	3.020E-09
	*LRP8* rs1288520	C/T	−0.569	0.230	−0.279	−2.473	0.014
	*LRP8* rs1288521	CC/CT/TT	0.327	0.096	0.227	3.409	0.001
	*LRP8* rs1288521	C/T	0.273	0.118	0.136	2.312	0.021
	*PCSK9* rs533375	CC/CT/TT	0.550	0.089	0.276	6.174	8.956E-10
	*PCSK9* rs533375	C/T	0.430	0.099	0.187	4.358	1.419E-05
	*PCSK9* rs584626	AA/AG/GG	0.421	0.199	0.129	2.116	0.035
	*PCSK9* rs584626	A/G	0.849	0.268	0.247	3.166	0.002
	*PCSK9* rs540796	GG/AG/AA	−0.603	0.221	−0.182	−2.730	0.006
	*PCSK9* rs540796	G/A	−1.051	0.341	−0.309	−3.083	0.002
	*AGBL4* A-A-G	Carriers/Non-carriers	0.890	0.109	0.205	8.133	9.903E-16
	*AGBL4* G-G-A	Carriers/Non-carriers	−0.319	0.054	−0.151	−5.942	3.632E-09
	*LRP8* A-A-G-T-C	Carriers/Non-carriers	0.129	0.051	0.065	2.509	0.012
	*LRP8* A-C-A-T-T	Carriers/Non-carriers	−0.522	0.127	−0.106	−4.099	4.409E-05
	*LRP8* G-A-G-C-C	Carriers/Non-carriers	0.511	0.068	0.191	7.550	8.322E-14
	*PCSK9* C-A-A-G	Carriers/Non-carriers	1.299	0.146	0.225	8.917	1.638E-18
	A-A-G-A-A-G-T-C-C-A-A-G	Carriers/Non-carriers	0.119	0.051	0.060	2.323	0.020
	A-A-G-G-A-G-C-C-C-A-A-G	Carriers/Non-carriers	0.538	0.067	0.203	8.049	1.919E-15
HDL-C	*PCSK9* rs533375	CC/CT/TT	−0.100	0.050	−0.109	−2.005	0.045
	*LRP8* rs6694764	G/A	0.185	0.091	0.194	2.029	0.043
	*LRP8* rs1288521	C/T	−0.130	0.062	−0.140	−2.097	0.036
	*PCSK9* rs533375	C/T	−0.133	0.052	−0.126	−2.586	0.010
	*LRP8* A-A-G-T-C	Carriers/Non-carriers	−0.059	0.026	−0.064	−2.276	0.023
LDL-C	*LRP8* rs1288520	C/T	0.224	0.112	0.255	1.999	0.046
	*LRP8* rs1288521	C/T	−0.129	0.057	−0.150	−2.248	0.025
	*LRP8* A-A-G-T-C	Carriers/Non-carriers	−0.059	0.024	−0.069	−2.464	0.014
	A-A-G-A-A-G-T-C-C-A-A-G	Carriers/Non-carriers	−0.057	0.024	−0.066	−2.375	0.018
ApoA1	*AGBL4* rs320017	A/G	−0.147	0.055	−0.347	−2.665	0.008
	*AGBL4* rs320018	A/G	0.146	0.065	0.346	2.236	0.026
	*PCSK9* rs540796	G/A	−0.153	0.074	−0.223	−2.064	0.039
	*PCSK9* C-G-A-A	Carriers/Non-carriers	0.049	0.021	0.061	2.282	0.023
ApoB	*AGBL4* rs320018	A/G	−0.158	0.079	−0.316	−1.992	0.047
	*AGBL4* rs320019	G/A	0.149	0.059	0.295	2.506	0.012
	*PCSK9* rs540796	G/A	0.240	0.090	0.294	2.660	0.008
	*AGBL4* A-A-G	Carriers/Non-carriers	−0.084	0.028	−0.080	−2.945	0.003
ApoA1/ApoB	*PCSK9* rs533375	CC/CT/TT	0.082	0.039	0.106	2.092	0.037
	*AGBL4* rs320018	A/G	0.365	0.123	0.454	2.966	0.003
	*AGBL4* rs320019	G/A	−0.205	0.092	−0.254	−2.231	0.026
	*PCSK9* rs584626	A/G	0.244	0.110	0.184	2.214	0.027
	*PCSK9* rs540796	G/A	−0.430	0.140	−0.328	−3.074	0.002
	*AGBL4* A-A-G	Carriers/Non-carriers	0.165	0.044	0.098	3.727	2.026E-04
	*LRP8* A-C-A-T-T	Carriers/Non-carriers	−0.137	0.051	−0.072	−2.704	0.007
	*PCSK9* C-A-A-G	Carriers/Non-carriers	0.185	0.059	0.083	3.126	0.002

HDL-C, high density lipoprotein cholesterol; LDL-C, low density lipoprotein cholesterol; Apo, apolipoprotein; *AGBL4*, the ATP/GTP binding protein-like 4 gene; *LRP8*, the LDL receptor related protein 8 gene; *PCSK9*, the Proprotein convertase subtilisin/kexin type 9 gene.
