# Supplementary material for: Integrative mutation, haplotype and G × G interaction evidence connects ABGL4, LRP8 and PCSK9 genes to cardiometabolic risk
Source: Sci Rep. 2016 Nov 17;6:37375. doi: 10.1038/srep37375 (PMC5112603; doi:10.1038/srep37375)
Supplement: Supplementary Information [file srep37375-s1.doc]

**Integrative mutation, haplotype and G×G interaction evidence connects ABGL4, LRP8 and PCSK9 genes to cardiometabolic risk**

Tao Guo^1^, Rui-Xing Yin^1^, Li-Mei Yao^1^, Feng Huang^1^, Ling Pan^1^, Wei-Xiong Lin^2^, De-Zhai Yang^2^ & Shang-Ling Pan^3^

**Supplemental Table 1** Characteristics of the *AGBL4, LRP8* and *PCSK9* mutations.

| **SNV ID (rs#)** | **HGVS Name** | **Chr:Position** | **Contig** | **Contig Pos** | **SNP to Chr** | **Allele** | **MAF/Minor** | **Map**  **Methods** |
| --- | --- | --- | --- | --- | --- | --- | --- | --- |
| ***AGBL4*** |  |  |  |  |  |  |  |  |
| rs320017 | NM_032785.3:c.952-18338T>C | 1: 48609323 | NT_032977.10 | 48023335 | Fwd | A | G=0.2422/1213 (1000 Genomes) | mapup |
| rs320018 | NM_032785.3:c.952-19314T>C | 1: 48610299 | NT_032977.10 | 48024311 | Fwd | A | G=0.2436/1220 (1000 Genomes) | mapup |
| rs320019 | NM_032785.3:c.952-19469C>T | 1: 48610454 | NT_032977.10 | 48024466 | Fwd | G | A=0.2696/1350 (1000 Genomes) | mapup |
| ***LRP8*** |  |  |  |  |  |  |  |  |
| rs6694764 | NM_033300.3:c.244+8447T>C | 1: 53318426 | NT_032977.10 | 52732438 | Fwd | A | A=0.3331/1668 (1000 Genomes) | mapup |
| rs1288519 | NM_033300.3:c.244+6396A>C | 1: 53320477 | NT_032977.10 | 52734489 | Rev | T | G=0.3702/1854 (1000 Genomes) | mapup |
| rs872315 | NM_033300.3:c.244+5185G>A | 1: 53321688 | NT_032977.10 | 52735700 | Rev | C | T=0.0292/146 (1000 Genomes) | mapup |
| rs1288520 | NM_033300.3:c.244+4912C>T | 1: 53321961 | NT_032977.10 | 52735973 | Rev | G | A=0.4401/2204 (1000 Genomes) | mapup |
| rs1288521 | NM_033300.3:c.244+4872G>A | 1: 53322001 | NT_032977.10 | 52736013 | Fwd | C | T=0.3317/1661 (1000 Genomes) | mapup |
| ***PCSK9*** |  |  |  |  |  |  |  |  |
| rs533375 | NM_174936.3:c.1180+174A>G | 1: 55057688 | NT_032977.10 | 54471700 | Rev | A | A=0.1412/707 (1000 Genomes) | mapup |
| rs584626 | NM_174936.3:c.1354+102C>T | 1: 55058311 | NT_032977.10 | 54472323 | Rev | C | C=0.1370/686 (1000 Genomes) | mapup |
| rs585131 | NM_174936.3:c.1355-56C>T | 1: 55058443 | NT_032977.10 | 54472455 | Rev | C | C=0.1308/655 (1000 Genomes) | mapup |
| rs540796 | NM_174936.3:c.1380A>G | 1: 55058524 | NT_032977.10 | 54472536 | Fwd | A | A=0.1456/17673 (ExAC)  A=0.1308/655 (1000 Genomes)  A=0.1822/2370 (GO-ESP) | mapup |

**Supplemental Table 2** The sequences of forward and backward primers of the *AGBL4, LRP8* and *PCSK9* mutations.

| **SNV** | **Primer sequence** | **Annealing temperature** | **PCR product** |
| --- | --- | --- | --- |
| ***AGBL4*** |  |  |  |
| rs320017 | ATGCCACTCCTCCACTTA | 48°C | 387bp |
|  | GCAACATAGCAATACCCT |  |  |
| rs320018 | CCCTCTAAGACTTGGTTT | 48°C | 387bp |
|  | GGTGTCTTAGTGACGGTC |  |  |
| rs320019 | CCAGTGCTCAGTGCCTAA | 48°C | 362bp |
|  | AATCCGTGACTCGTGACC |  |  |
| ***LRP8*** |  |  |  |
| rs6694764 | CCTGGCTTTGCTTTCTGT | 52°C | 216bp |
|  | GTCTTGCCGTTACTACTT |  |  |
| rs1288519 | AGAGGCTGGTGCTTGGAG | 54°C | 422bp |
|  | AATGGTGCCCTACTGACG |  |  |
| rs872315 | GGGGAAGAAATGAGTCAAAC | 53.5°C | 461bp |
|  | TAACCTGGTGCTGGGAGA |  |  |
| rs1288520 | GCAGTGGGTGAGGTTGTT | 54°C | 422bp |
|  | AAGGGAAAGTCTTCAGCAAA |  |  |
| rs1288521 | AAGGGAAAGTCTTCAGCAA | 52°C | 450bp |
|  | CTGGTAAGCAGGAGGTGTT |  |  |
| ***PCSK9*** |  |  |  |
| rs533375 | TCCCTCCTCCAAGATGCC | 56°C | 464bp |
|  | CCACCTCCTCACCTTTCC |  |  |
| rs584626 | CCTGGAGAAACTGGAGCA | 54°C | 471bp |
|  | AGGACCAGCGGGTACTGA |  |  |
| rs585131 | TCCTGGAGAAACTGGAGCA | 56°C | 472bp |
|  | AGGACCAGCGGGTACTGA |  |  |
| rs540796 | GAGGATGACGCCACCTTA | 54°C | 315bp |
|  | CCTGGAGAAACTGGAGCAG |  |  |
